# The Clinical Society of the Buffalo General Hospital

**Published:** 1901-01

**Authors:** E. L. Ruffner


### THE CLINICAL SOCIETY OF THE BUFFALO GENERAL HOSPITAL.

Reported by E. L. RUFFNER, M. D., Secretary pro tem.

FOR a closer *esprit de corps* in the clinical study of cases and the knowledge to be derived from such study the staff of the Buffalo General Hospital, their assistants and the clinical instructors in medicine have organised a clinical society of the Buffalo General Hospital.

The meeting was called to order, on Friday evening, November 2, 1900, at 8.15, in the medical clinic room.

Dr. Irving M. Snow was elected chairman; Dr. E. L. Ruffner was appointed secretary pro tem.

Dr. Roswell Park presented from his wards: (1) Man; severe burn from being caught in live electric wire, of neck and arms; skin grafting will be performed later. (2) Man; epithelioma of right half of tongue and floor of mouth; operation having removed half of the tongue. (3) Tumor of abdomen—adeno-carcinoma (?) first on medical service of Dr. Cary. Exploratory laparatomy made by Dr. Park, and condition did not warrant further procedure. (4) Man; accident—injury to spine, paralysis lower extremities, bladder, etc; clot of blood removed between first and second lumbar vertebræ, from chord. Bladder improved; some improvement in sensation of limbs. (5) Woman; movable kidney (right)—removed.

Dr. Charles Cary then presented (1) Woman; malaria—æstivo-autumnal—not a resident of Buffalo. Had been treated along lines of typhoid fever, with plunges, until blood examination showed filaria. (2) Man; aneurism of the arch of the aorta. (3) Double lobar pneumonia with icterus.

Dr. Charles G. Stockton then presented (1) Mulatto woman; high temperature for two weeks. Transferred to medical service by Dr. Crockett, with report that pelvic organs did not account for temperature. Blood does not show typhoid, malaria leucocytosis (?) anemia. Suspicious rash on back; now on specific treatment; diagnosis not yet made. (2) Man; esophageal stenosis; sixteen inches from teeth; probably carcinomatous. (3) Man; severe case of vomiting prolonged, with gastro-ptosis and dilated colon from obstruction. Obstruction not ascertained yet. Man very sluggish mentally.

Dr. H. U. Williams then presented for inspection a specimen of trichina spiralis in muscle. Cultures of amebæ from colon, in case of amebic dysentery, culture of B. Friedlander, amebic ulceration of colon.

Dr. H. R. Gaylord then presented under microscope a number of rare conditions.

Twenty-five members present.

